# TRAIL promotes epithelial-to-mesenchymal transition by inducing PD-L1 expression in esophageal squamous cell carcinomas

**DOI:** 10.1186/s13046-021-01972-0

**Published:** 2021-06-24

**Authors:** Huanyu Zhang, Guohui Qin, Chaoqi Zhang, Huiyun Yang, Jinyan Liu, Hongwei Hu, Peng Wu, Shasha Liu, Li Yang, Xinfeng Chen, Xueke Zhao, Lidong Wang, Yi Zhang

**Affiliations:** 1 Biotherapy Center & Cancer Center, the First Affiliated Hospital, Zhengzhou University, 1 Jianshe East Road, Henan 450052 Zhengzhou, China; 2 State Key Laboratory of Esophageal Cancer Prevention & Treatment, Henan 450052 Zhengzhou, China; 3 Department of Thoracic Surgery, National Cancer Center/National Clinical Research Center for Cancer/Cancer Hospital, Chinese Academy of Medical Sciences and Peking Union Medical College, 100021 Beijing, China; 4 Henan Key Laboratory for Tumor Immunology and Biotherapy, 450052 Zhengzhou, China; 5 School of Life Sciences, Zhengzhou University, 450052 Zhengzhou, China

**Keywords:** TRAIL, Tumorigenesis, EMT, PD-L1, Immunotherapy

## Abstract

**Background:**

Tumor necrosis factor-associated apoptosis-inducing ligand (TRAIL) was initially considered an immunity guard; however, its function remains controversial. Besides immune cells, lung and colon cancer cells have also been reported to express TRAIL, which can promote tumor invasion and metastasis. However, the biological function and underlying mechanism of action of TRAIL in esophageal squamous cell carcinoma (ESCC) remain poorly elucidated.

**Methods:**

The ESCC cells stemness, migration, and proliferation ability was assessed by sphere formation, Transwell, and CCK8 assay. The stemness- and epithelial-mesenchymal transition (EMT)- related genes expression levels were analyzed by Western blot and RT-qPCR. The signal activation was conducted by Western blot. The xenograft mouse experiments and lung metastasis model were performed to confirm our findings in vitro.

**Results:**

Herein, we found that TRAIL is a negative predictor in patients with ESCC. To further investigate the biological function of TRAIL, we established TRAIL knockdown and overexpression ESCC cell lines and found that TRAIL induced EMT and promoted tumor aggressiveness. Furthermore, we demonstrated that TRAIL- overexpressing cells upregulated PD-L1 expression, which was dependent on the p-ERK/STAT3 signaling pathway. We obtained similar results when using recombinant human TRAIL. Finally, we validated the biological role and mechanism of action of TRAIL in vivo.

**Conclusions:**

These findings demonstrate that TRAIL promotes ESCC progression by enhancing PD-L1 expression, which induces EMT. This may explain the failure of TRAIL preclinical trials.

**Supplementary Information:**

The online version contains supplementary material available at 10.1186/s13046-021-01972-0.

## Introduction

Tumor necrosis factor-related apoptosis inducing ligand (TRAIL) is a member of tumor necrosis factor superfamily (TNFSF) and is encoded by *TNFSF10*. To function, TRAIL must interact with its receptors. To date, five TRAIL receptors have been identified: TRAIL-R1 (DR4), TRAIL-R2 (DR5), TRAIL-R3 (DCR4), TRAIL-R4 (DCR5), and osteoprotegerin (OPG) [1–3]. Initially, TRAIL was found expressed on various immune cells, such as T cells, B cells, and NK cells, and involved in both innate and adaptive immune responses [4–6]. Recently, TRAIL was identified with a diversity of function in the tumor microenvironment. Recently, TRAIL was identified with a diversity of functions in the tumor microenvironment. On the one hand, TRAIL induces tumor cells apoptosis via binding to its receptors DR4/5 [7, 8]. On the other hand, tumor cells could recruit M2 by expressing TRAIL, remodeling the tumor microenvironment and promoting tumor progression [9]. However, how TRAIL regulates tumor cell epithelial-mesenchymal transition (EMT) to affect tumor cell metastasis has not been investigated.

Tumor invasion and metastasis is a complex multi-step cascade of responses that is constantly changed by the interaction of tumor cells with the host microenvironment [10], and they are the main reasons for the poor prognosis in clinic. Among the various factors, inflammatory cytokines and immunosuppressive cells were considered the key points of EMT and distant metastasis [10, 11]. Many factors have been reported to induce EMT in a variety of cancers. Recently, the correlation between PD-L1 expression and EMT has attracted increasing attention [12]. It has been demonstrated that PD-L1 promotes EMT in esophageal cancer, pancreatic cancer, breast cancer, and non-small cell lung cancers, leading to tumor progression [13–16]. Meanwhile, it has been reported that TRAIL and PD-L1 can be expressed simultaneously on tumor cells [17]. However, the factors inducing the abnormal expression of PD-L1 are less defined. Esophageal squamous cell carcinoma (ESCC) is one of the most aggressive malignant tumors, and with various therapies, the 5-year survival rate of ESCC is only 15–25 % [18, 19]. Therefore, revealing the underlying mechanism of ESCC distant metastasis may be fundamental for elevating clinical efficacy, but studies on blocking EMT to inhibit ESCC progression currently lack effective targets.

In this study, we found that TRAIL was a poor predictor in patients with ESCC. To further explain the phenomenon, we explored the source of TRAIL in the ESCC tumor microenvironment and evaluated its function in vitro and in vivo. We found that TRAIL was expressed by ESCC cells and can induce EMT by regulating PD-L1 expression. Knocking down TRAIL (*TNFSF10)* in ESCC cells suppressed EMT through inhibition of ERK/STAT3 signaling. It is evident that TRAIL can promote ESCC progression by influencing EMT, thus TRAIL has the potential to act as a key molecule to target EMT and inhibit ESCC progression in the future.

## Materials and methods

### Human clinical samples

A total of 83 paired tumor and corresponding nontumor tissues (located more than 3 cm away from tumor tissues) were freshly obtained from patients with ESCC (diagnosed in 2017–2018) at the department of Thoracic Surgery of the First Affiliated Hospital of Zhengzhou University (Zhengzhou, China). All patients provided written informed consent before tissues or associated clinical information were collected, and this research was approved by the Institutional Ethical Committee of the First Affiliated Hospital of Zhengzhou University (2018-KY-92). Written informed consent was obtained from each patient with available follow-up information prior to participation.

### Tissue microarray construction

A total of 98 patients with primary esophageal squamous cell carcinoma, who received radical esophagectomy without neoadjuvant chemoradiotherapy in National Cancer Center/National Clinical Research Center for Cancer/Chinese Academy of Medical Sciences and Peking Union Medical College between January 2019 and December 2019, were included in this retrospective study. These cases were non-consecutive cases which had complete follow-up data. All tumor samples were fixed in 10 % neutral buffered formalin for 12–48 h and embedded in paraffin. Tissue microarrays (TMAs) were constructed from three 1.0-mm cores of tumor tissue and three 1.0-mm cores of normal epithelium from each case using a Manual Tissue Arrayer (MTA-1, Beecher Instruments, Silver Spring, MD).

### Cell lines and cell culture

Human ESCC cell lines (KYSE150, KYSE70, EC1, EC109, EC9706, TE1, and TE7), normal esophageal epithelial cell line (HET-1α) and human embryonic kidney epithelial 293T cells (293T) were obtained from the Cell bank Shanghai Institutes for Biological Sciences of the Chinese Academy of Sciences. ESCC cells and HET-1α were cultured in RPMI 1640 medium supplemented with 10 % FBS and 1 % cyan streptomycin mixture. The virus packaging cell line 293T cultured in DMEM supplemented with 10 % FBS and 1 % cyan streptomycin mixture. Cells were cultured in an incubator with 5 % CO_2_ and 37 °C.

### Cell proliferation assays

A total of 1 × 10^3^ cells per well were plated in 96-well plates for cell proliferation assays. We set up five replicate wells for each group per experiment. Each experiment was repeated three times. CCK-8 (10 µL; CK04-500; Dojindo, Kumamoto, Japan) was added at 0, 24, 48, and 96 h and incubated at 37 °C for 2 h. Absorbance was detected at 450 nm using an enzyme-labeled instrument (Multiskan MK3; Thermo Fisher Scientific, Waltham, MA, USA).

### Transwell invasion assays

A Transwell plate with an 8 μm membrane was used for testing tumor cell migration. Tumor cells (1 × 10^5^) without resuspension in serum were inoculated into the upper chamber, and 600 µL of complete medium was added to the lower chamber. The cells were cultured in a cell incubator for 24 h, fixed with 4 % paraformaldehyde at room temperature for 30 min, and stained with 0.1 % crystal violet solution for 30 min. After taking pictures, the number of cells in each field was counted and a statistical chart was drawn.

### Cell sphere experiment

The cell density was adjusted to 2 × 10^3^ cells/mL, after which a 2-mL cell suspension was added to a 24-well low adhesion plate. After 5 days, the cell spheres were observed and counted under a microscope.

### RNA isolation and real-time PCR

Total RNA was isolated using RNAiso Plus (Takara Bio, Shiga, Japan) and reverse-transcribed using PrimeScript™ II 1st Strand cDNA Synthesis Kit (Takara Bio) according to manufacturer’s instructions. RT-qPCR was performed using SYBR Green (BCS, Australia). GAPDH was used for normalization of data. The data were analyzed using the 2^ΔΔCt^ method. All primers are listed in Supplementary Table 1.

### Western blotting

Protein samples were isolated from cells and animal tissues, after which they were resolved by SDS-polyacrylamide gel electrophoresis and transferred to a 0.2-µm nitrocellulose membrane (GE Healthcare, Chicago, IL USA). The blots were blocked with 5 % non-fat dry milk in Tris-buffered saline for 2 h at room temperature and immunoblotted with the appropriate primary antibody at 4 °C overnight. The next day, the blots were incubated for 1 h at room temperature with secondary antibodies. The Fusion FX7 ECL western blot system (Vilber Lourmat, Marne-la-Vallée, France) was used to visualize the protein signals. All antibodies are listed in Supplementary Table 1.

### Immunohistochemistry

Tissue paraffin sections of tumors were deparaffinized in xylene and rehydrated. Slides were blocked and incubated with primary antibody at 4 °C overnight. The next day, slides were treated with biotinylated IgG secondary antibodies, followed by detection with the DAB substrate kit (Zsbio, Beijing, China). Images were acquired using a microscope (Olympus, IX73). Staining was evaluated based on intensity (negative = 0; weak = 1; moderate = 2; and high = 3) of immunostaining and density (0 % = 0; 1–40 % = 1; 41–75 % = 2; >76 % = 3) of positive tumor cells. All antibodies are listed in Supplementary Table 1.

### Immunofluorescence

Cells (5 × 10^4^) were seeded into 24-well plates, fixed in 4 % paraformaldehyde, and permeabilized with 0.1 % Triton X-100. Next, 5 % goat serum was used to block cells for 30 min, after which the cells were incubated with primary antibodies at 4 °C overnight. The cells were then incubated with secondary antibodies (BioLegend, San Diego, CA) for 1 h and counterstained with 4′,6-diamidino-2-phenylindole (DAPI; BioLegend). Images were acquired using a fluorescence microscope and analyzed using ImageJ software (National Institutes of Health, Bethesda, MD). All antibodies are listed in Supplementary Table 1.

### Plasmid construction and stable transfection

The hU6-MCS-Ubiquitin-EGFP-IRES-puromycin plasmids were purchased from GenePharma (Shanghai, China). High-titer lentivirus was packaged in HEK293T cells using transfection kits (Polyplus transfection #20Y0305L9). Viral supernatants were collected at 48 h after transfection, filtered, and added to ESCC cells in the presence of 10 µg/mL polybrene for 24 h. Finally, the transfection efficiency was checked by RT-qPCR and flow cytometer.

### siRNA transfection

The cells were cultured in 6-well plates at 3 × 10^5^ cells per well. The total amount of transfection mixture for each well consisted of 200 µL jetPRIME buffer, 10 µL siRNA, 4 µL jetPRIME reagent. The mixture was then centrifuged with a palm centrifuge and incubated at room temperature for 15 min. The above transfection mixture system was evenly added into a hole. After 60 h, the cells were collected for subsequent experiments.

### Flow cytometry analysis

Cells (1 × 10^6^) were stained (specific antibody information is listed in Supplementary Table 1), vortexed, and incubated in a refrigerator at 4 °C in the dark for 15 min. After washing with 1 mL flow buffer (PBS with 2 % serum), the samples were analyzed using a BDFACS Canto II Cell Analyzer (BD Biosciences, Franklin Lakes, NJ). FlowJo software (TreeStar, Inc., Ashland, OR) was used for data analysis.

### Animal experiments

Female athymic BALB/c nude mice (4–6 weeksold) and severe combined immune deficiency beige (SCID/Beige) mice (4-6 weeks old) were purchased from Vital River Laboratory Animal Technology Company (Beijing, China). All animal procedures were conducted in accordance with the Guide for the Care and Use of Laboratory Animals and were approved by the Institutional Animal Care and Use Committee of the First Affiliated Hospital of Zhengzhou University. BALB/c nude mice were used for the subcutaneous xenograft experiments, and SCID/Beige mice were used for the lung metastasis experiment. For the in vivo xenograft experiment, BALB/c nude mice were subcutaneously injected with 5 × 10^6^ tumor cells. After 21 days, the mice were sacrificed. Tumor size was measured using calipers every two days and tumor volume was calculated using the formula V = (width^2^ ×length)/2. For the Lung metastasis model, 1 ×106 tumor cells were injected into the tail vein of SCID/Beige mice and treated with anti-PDL1 antibody (10mg/kg, twice a week, intraperitoneal injection). The mice were sacrificed and the lungs were harvested and fixed with paraformaldehyde after 5 weeks for picric acid staining. All the xenograft mouse model experiments were conducted in the Henan Key Laboratory for Pharmacology of Liver Diseases, and the animals’ certificate were approved by the ethics committee of Henan Key Laboratory for Pharmacology of Liver Diseases (Approval No. 2019-41).

### The Cancer Genome Atlas (TCGA) database analysis

mRNA sequencing data of patients with ESCC and healthy individuals were downloaded from TCGA database. Fifteen immunosuppressive molecules, such as PD-L1 (*CD274*), were selected for correlation analysis with TRAIL. Patients with ESCC were divided into TRAIL^high^ and TRAIL^low^ groups according to TRAIL mRNA expression.

### Statistical analysis

For experiments with two groups, statistical significance was determined by Student’s *t*-test. For experiments with more than three groups, analysis of variance followed by post-hoc pairwise comparisons was performed. Kaplan-Meier analysis was used to determine the difference in overall survival. Data shown are the means ± SD of at least three independent experiments performed in duplicate. *P* values < 0.05 were considered statistically significant.

## Results

### Abnormal expression of TRAIL in ESCC is negatively correlated with patient clinical outcomes

**Fig. 1:**
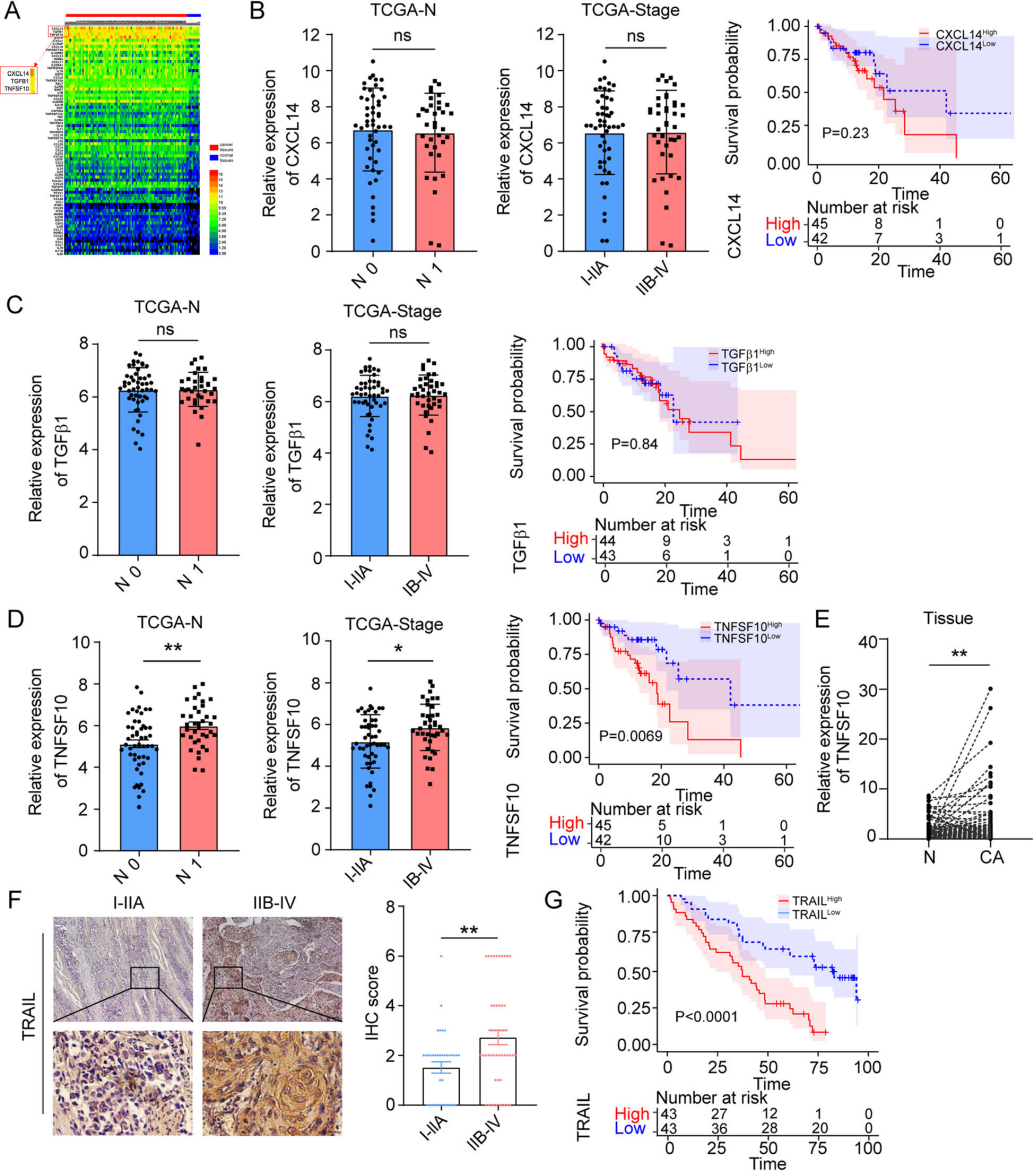
High expression of TRAIL in ECSS is positively correlated with lymph node metastasis, stage, and clinical prognosis of patients. **a** Comparison of differential genes in ESCC tissues (*n* = 93) and normal esophageal tissues (*n* = 11) in TCGA database. **b-d** correlation of *TGFB1*, *CXCL14*, and *TNFSF10* expression with metastasis, staging and clinical prognosis in patients in TCGA database. **e** The expression of TRAIL in cancer tissues is higher than in adjacent normal tissues at the mRNA level (*n* = 83). **f** Immunohistochemical results showed higher expression of TRAIL in advanced tumors than in early tumor tissues. **g** High TRAIL expression is negatively associated with clinical survival. NC, negative control, **P< 0.01, *P < 0.05 (unpaired *t*-test)

To reveal the expression patterns of inflammatory factors and the corresponding receptors in ESCC tumor and normal tissues, we analyzed TCGA RNA-seq database. We found that *CXCL14*, *TGFβ1*, and *TNFSF10* were highly expressed in tumor tissues compared with adjacent normal samples (Fig. 1a). Next, we analyzed the relationship between these three genes and patient clinical parameters. *CXCL14* and *TGFβ1* were not correlated with lymph node metastasis, staging, or overall survival (Fig. 1b, c). Only *TNFSF10* had a significant positive relationship with lymph node metastasis and staging, and patients with high *TNFSF10* levels exhibited poor prognosis (Fig. 1d). To further verify this, we analyzed TRAIL expression in 83 patients with ESCC at both the mRNA and protein levels. A similar trend was observed, and TRAIL was highly expressed in tumor tissues compared with adjacent normal samples (Fig. 1e, f). In addition, TRAIL was significantly increased in advanced stages (IIB–IV) compared with the early stages (I–IIA; Fig. 1f, Supplementary Fig. 1a) and was negatively correlated with overall survival of patients with ESCC (Fig. 1g). Together, these data suggest that TRAIL accumulates in ESCC tumor sites and is negatively correlated with patient survival.

### TRAIL is expressed by ESCC cells and knocking it down reduces ESCC cell migration in vitro

**Fig. 2:**
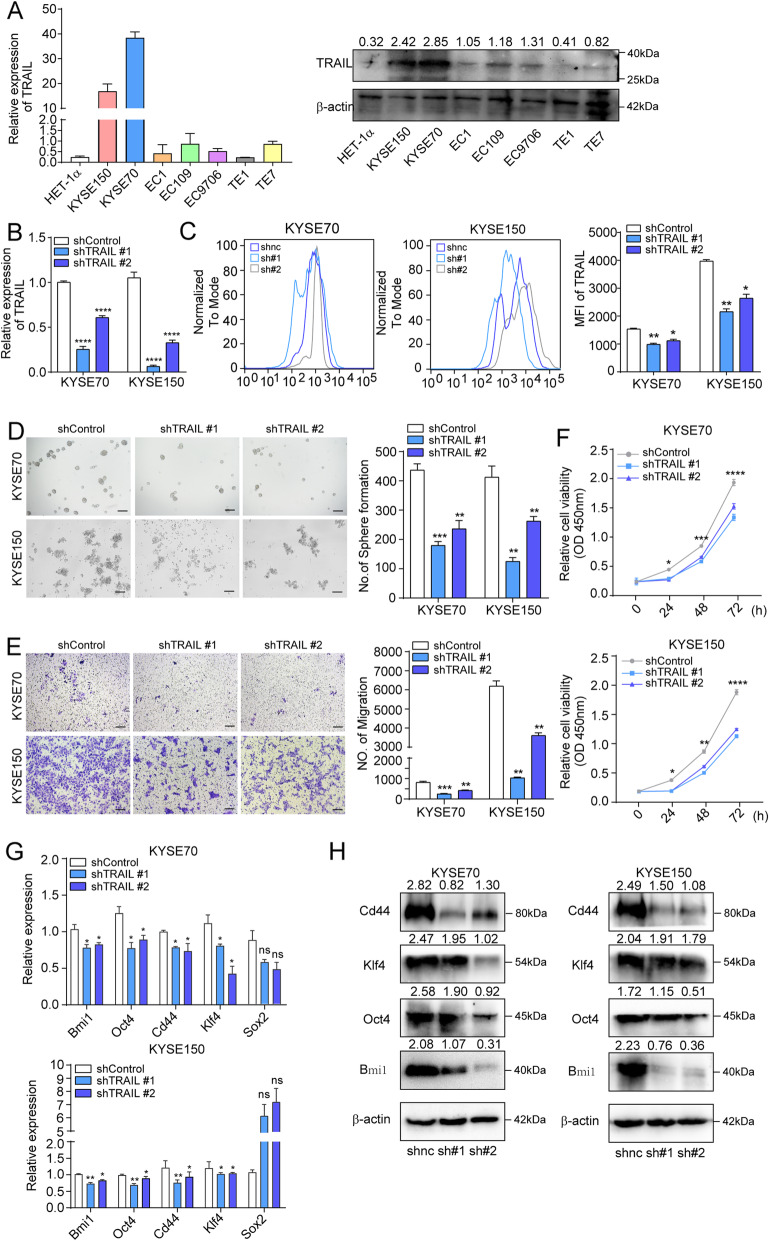
Knockdown of TRAIL inhibits ESCC cell migration, invasion, and proliferation in vitro. **a** TRAIL is differentially expressed in different ESCC samples as evidenced by RT-qPCR and western blotting. **b, c** The TRAIL knockdown effects in KYSE70 and KYSE150 cells were confirmed by RT-qPCR and flow cytometry. GAPDH was used as an internal loading control. **d** The effect of TRAIL on sphere formation of KYSE70 (top) and KYSE150 (bottom) cells. Representative images and quantitative analysis are shown in the left and right panels, respectively. **e** Transwell assay of TRAIL knockdown and control KYSE70 and KYSE150 cells. Representative images and quantitative analysis are shown in the left and right panels, respectively. **f** Cell proliferation determined by the CCK-8 assay with/without TRAIL knockdown in KYSE70 (top) or KYSE150 (bottom) cells. **g, h** Gene and protein expression of cell stemness markers in KYSE70 (top) or KYSE150 (bottom) cells. NC, negative control. The data are presented as the mean ± SEM. ****P< 0.0001, ***P < 0.001, **P < 0.01, *P < 0.05 (unpaired *t*-test)

Initially, TRAIL was reported to be expressed by immune cells and that it defends against pathogens and self-antigens. However, TRAIL was found expressed by colon and lung cancers and a contributor to tumor progression in recent years [9]. Herein, TRAIL was found negatively correlated with patient prognosis, and immunohistochemistry data revealed that TRAIL was mainly expressed around the tumor cells. Thus, we hypothesized that ESCC cells may be the source of TRAIL. To determine whether ESCC cells express TRAIL, we examined TRAIL expression in seven human ESCC cell lines at different stages of differentiation ( KYSE150, KYSE70, EC1, EC109, EC9706, TE1 and TE-7) and normal esophageal epithelial cell line (HET-1α) at the mRNA and protein levels (Fig. 2a). TRAIL expression was found heterogeneous in ESCC cell lines, with KYSE150/KYSE70 cells exhibiting the highest expression levels (Fig. 2a).

To evaluate the possible biological function of TRAIL in ESCC, we silenced TRAIL in KYSE70 and KYSE150 cells using siRNAs. After verifying the silencing efficiency by RT-qPCR (Supplementary Fig. 1b), sphere formation, Transwell, and CCK8 assays were performed. The data showed that knocking down TRAIL significantly reduced the ability of cells to form spheroids, migrate, and proliferate (Supplementary Fig. 1c–e). Moreover, we analyzed stemness-related gene expression (Bmi1, Oct4, and Klf4) and found that TRAIL silencing decreased their gene expression levels (Supplementary Fig. 1f). These results suggest that TRAIL may enhance the stem properties of ESCC. To investigate this, we knocked down TRAIL using short hairpin RNAs (shRNAs); knockdown efficiency was verified at both the mRNA and protein levels (Fig. 2b, c), after which sphere formation, Transwell, and CCK8 assays were performed. Similarly, knocking down TRAIL reduced ESCC cell migration, spheroid formation, and proliferation ability (Fig. 2d–f). Moreover, expression of the stemness-related genes Bmi1, Oct4, Cd44, and Klf4 significantly declined in TRAIL-knockdown KESE70 and KYSE150 cells; the change in Sox2 expression levels were not significant (Fig. 2g, h). Together, these results suggest that ESCC cells express TRAIL and that TRAIL may promote tumor stemness.

### Overexpression of TRAIL promotes ESCC cell migration, invasion, and proliferation in vitro

**Fig. 3:**
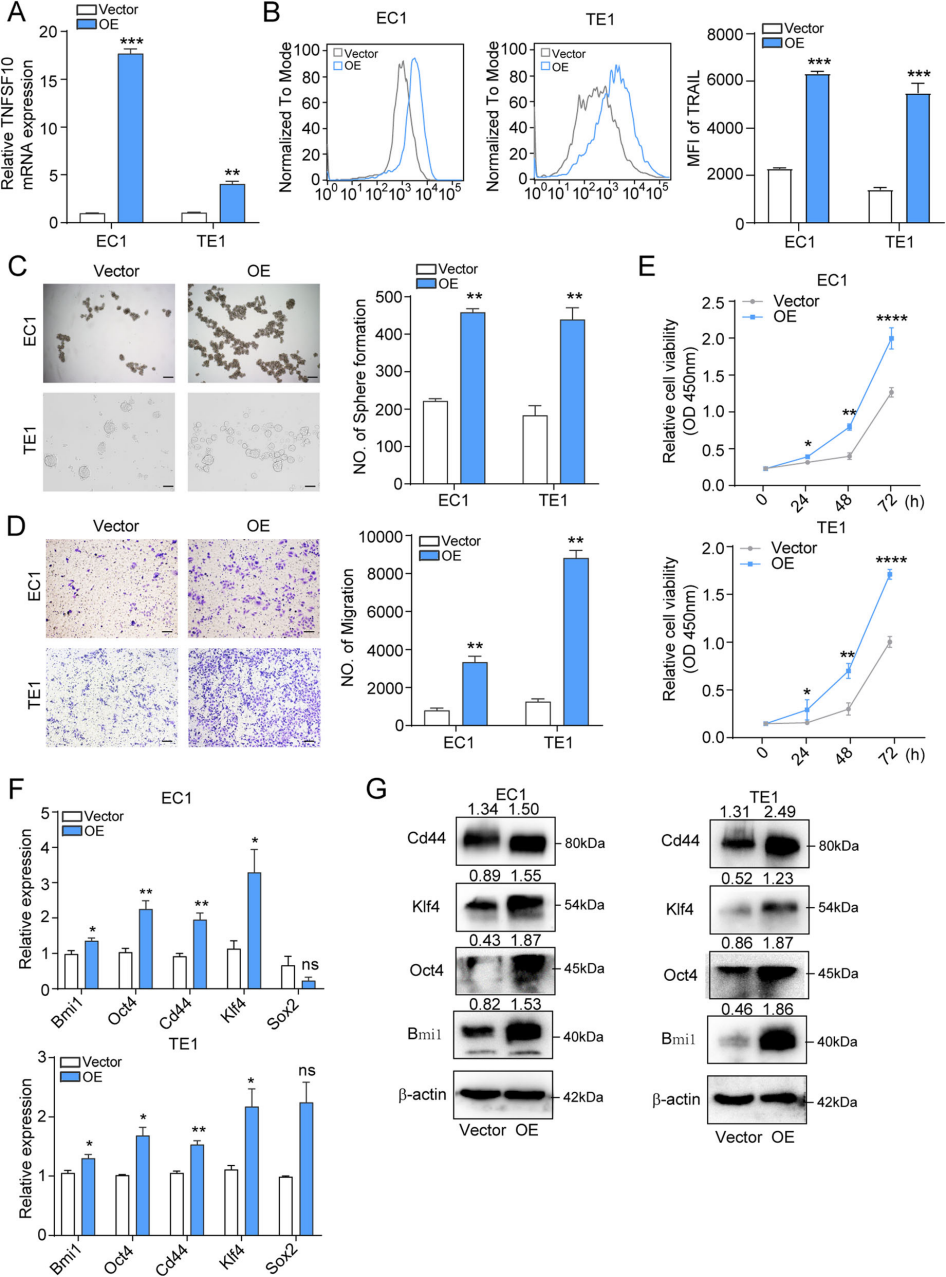
Overexpression of TRAIL promotes ESCC cell migration, invasion, and proliferation. **a, b** TRAIL overexpression effects in EC1 and TE1 cells were confirmed by RT-qPCR (left) and flow cytometry (right). **c, d** Sphere formation and transwell assays of TRAIL overexpression and control EC1 and TE1 cells. Representative images and quantitative analysis are shown in the left and right panels, respectively. **e** CCK-8 assay to determine cell proliferative capacity of EC1 (top) and TE1 (bottom) cells. **f, g** RT-qPCR and western blot of cell stemness-related genes. NC, negative control. The data are presented as the mean ± SD. ****P < 0.0001, ***P < 0.001, **P < 0.01, *P < 0.05 (unpaired *t*-test)

We next stably overexpressed TRAIL in EC1 and TE1 cells, which showed the lowest TRAIL levels. After verifying the efficacy at both the mRNA and protein levels (Fig. 3a, b) we performed sphere formation, Transwell, and CCK8 assays. And TRAIL overexpression significantly enhanced EC1/TE1 cell sphere formation (Fig. 3c), migration (Fig. 3d), and proliferation (Fig. 3e) abilities. As for the stemness-related genes, the expression was significantly increased in TRAIL overexpressed cells compared with the control group (Fig. 3f, g). We further used recombinant human TRAIL (rh-TRAIL) to verify the results. Similar to the overexpression system, rh-TRAIL treatment significantly increased the expression of the stemness-related markers CD271, CXCR4, and Bmi1 (Supplementary Fig. 2a–c). Additionally, rh-TRAIL significantly promoted cellular migration and sphere formation (Supplementary Fig. 2d, e). Taken together, our data indicate that TRAIL facilitates the migration, invasion, proliferation, and stemness of ESCC cells.

### TRAIL promotes EMT of ESCC cells

**Fig. 4:**
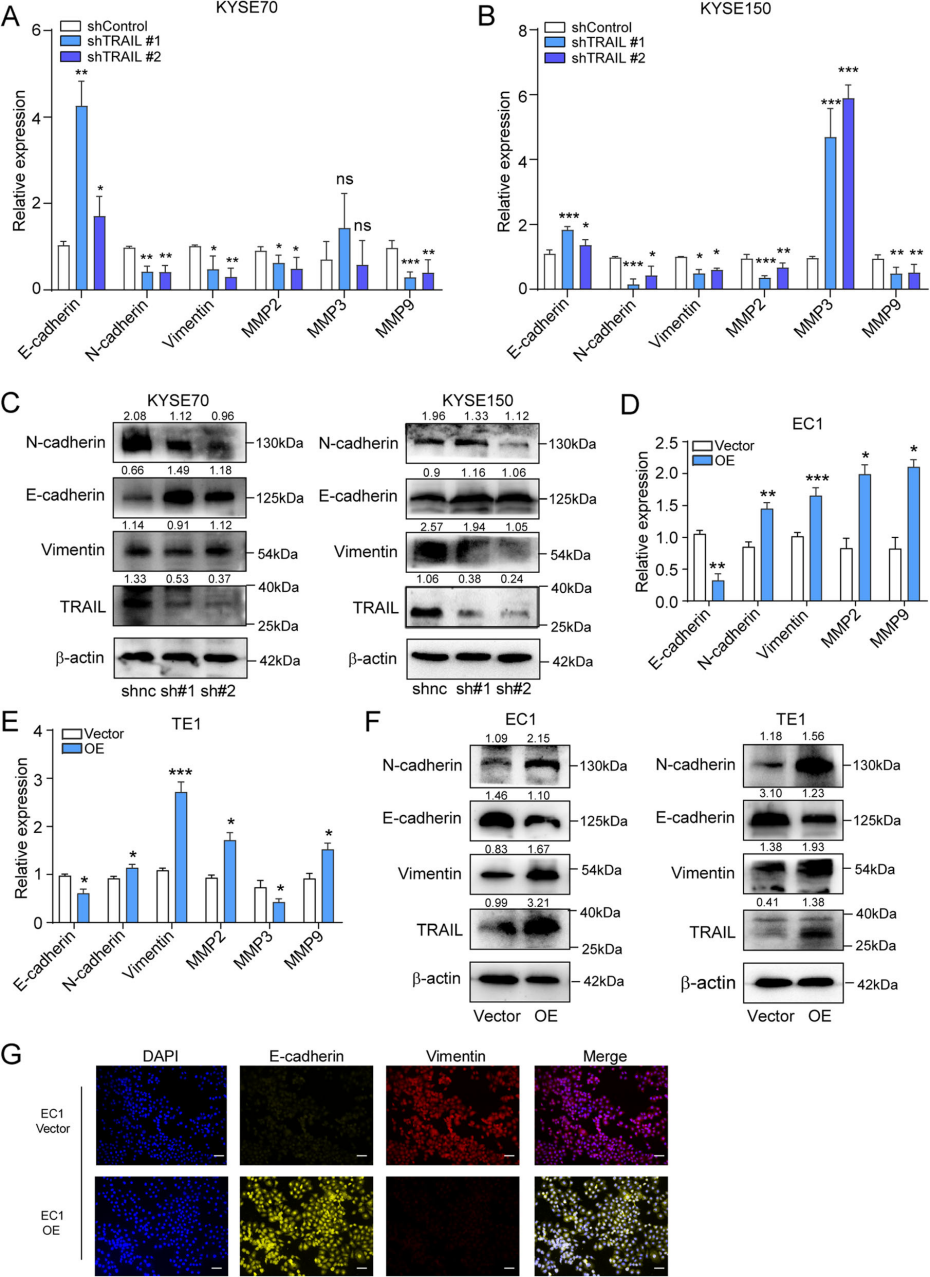
TRAIL facilitates epithelial-mesenchymal transition (EMT) by regulating the expression of EMT-related proteins. **a, b** RT-qPCR of EMT-related genes in TRAIL-knockdown KYSE70 and KYSE150 cells. **c** Western blotting of EMT-related proteins in TRAIL-knockdown KYSE70 or KYSE150 cells and the respective controls. **d–f** mRNA and protein levels of EMT-related genes in TRAIL-overexpressing EC1 and TE1 cells. **g** Immunohistochemistry of vimentin and E-cadherin expression in TRAIL-overexpressing EC1 cells and respective controls. NC, negative control. The data are presented as the mean ± SEM. ***P < 0.001, **P < 0.01, *P < 0.05 (unpaired *t*-test)

Previous studies have shown that tumor distant metastasis and migration mainly result from EMT [20] and that TRAIL regulates EMT in breast cancer [21]. Given that TRAIL was found expressed by ESCC cells, which promoted their migration and stemness, we investigated whether TRAIL promotes ESCC stemness and migration in an EMT-dependent manner. We examined the main markers associated with EMT and found that knocking out TRAIL significantly increased E-cadherin expression and markedly reduced N-cadherin, vimentin, MMP2, and MMP9 levels (Fig. 4a, b). Of note, MMP3 was significantly elevated in shTRAIL-KYSE150 cells, but not in shTRAIL-KYSE70 cells (Fig. 4a, b). During the initial EMT step, cancer cells secrete MMPs to degrade collagen and fibrin [22]. MMP2/3/9 can degrade collagen and fibrin, which may explain why these two cell lines showed different MMP expression patterns. The TRAIL-induced modulation of EMT-related genes (E-cadherin, N-cadherin, and vimentin) were further validated at the protein level by western blotting (Fig. 4c). Similar results were obtained after using siRNA to knockdown TRAIL (Supplementary Fig. 3a, b). We also found that TRAIL showed a significant negative correlation with E-cadherin and a significant positive correlation with N-cadherin, and vimentin by tissue microarray. (Supplementary Fig. 3e).

On the other hand, when TRAIL was overexpressed in EC1 and TE1 cells, we observed the opposite trends at both the mRNA (Fig. 4d, e) and protein levels (Fig. 4e–g). Adding human recombinant TRAIL increased vimentin and N-cadherin expression levels, reduced E-cadherin expression (Supplementary Fig. 3c, d). Taken together, these results indicate that TRAIL promotes EMT of ESCC cells, which may underly the promotion of ESCC progression by TRAIL.

### TRAIL activates the ERK/STAT3 signaling pathway to induce PD-L1 expression in ESCC

**Fig. 5:**
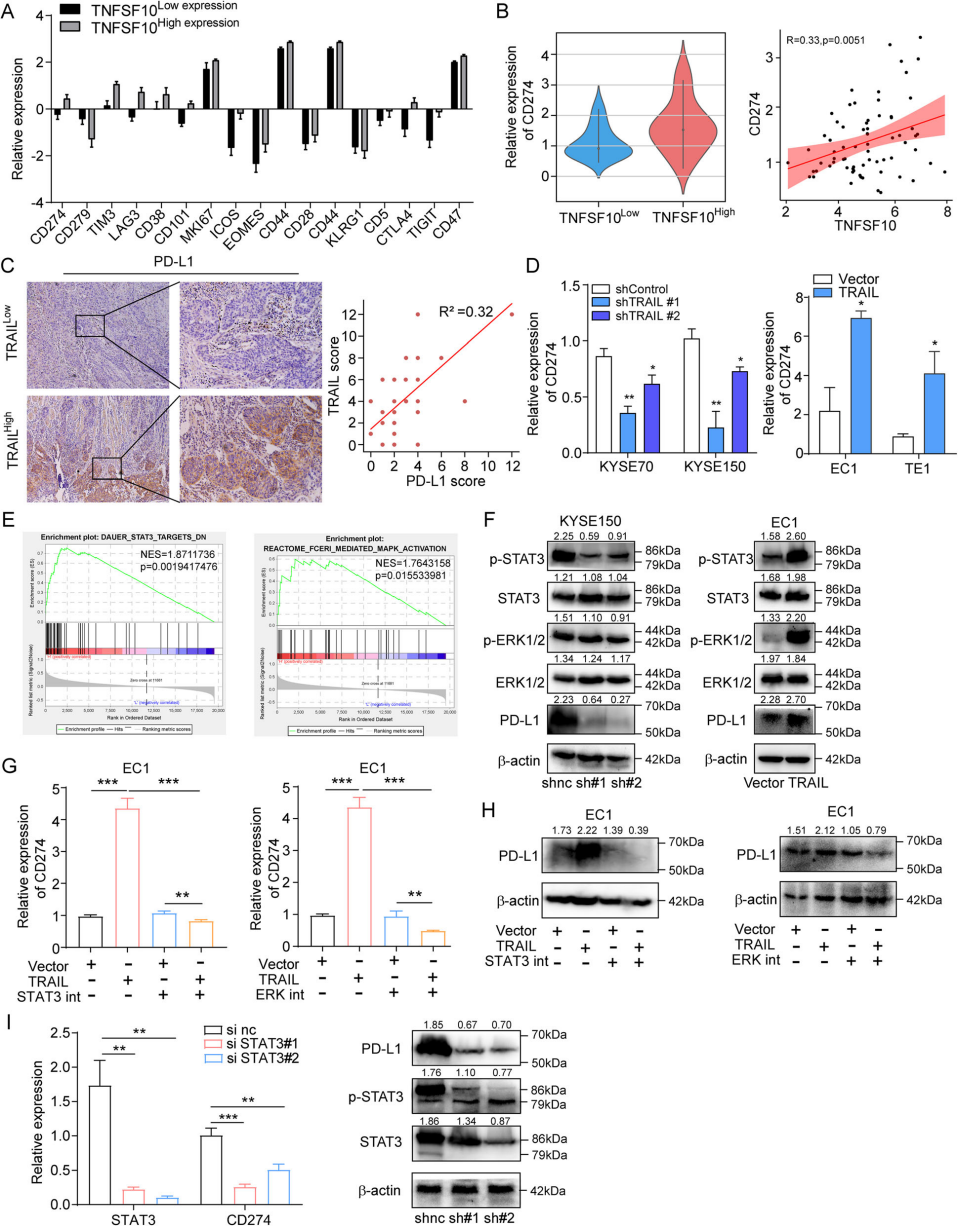
TRAIL promotes PD-L1 expression by upregulating the p-ERK/STAT3 signaling pathway. **a, b** Analysis of *TNFSF10* high- and low-expression genes; *TNFSF10* and *CD274* expression was significantly positively correlated in TCGA database. **c** TRAIL and PD-L1 expression was significantly positively correlated in human ESCC tissues (*n* = 20), as evidenced by immunohistochemistry. **d** RT-qPCR of *CD274* mRNA levels in TRAIL-knockdown (left) and overexpressed (right) ESCC cell lines. **e** KEGG enrichment analysis of differentially expressed genes. **f** Western blotting of STAT3, p-STAT3, ERK1/2, p-ERK1/2, PD-L1, and b-actin expression in TRAIL-knockdown KYSE150 or TRAIL-overexpressed EC1. **g, h** RT-qPCR and western blotting of PD-L1 mRNA and protein levels after adding STAT3 or ERK inhibitors for 24 h; GAPDH and b-actin were used as control for RT-qPCR and western blotting, respectively. **i** Silenced STAT3 in EC1 cells overexpressing TRAIL and the expression of PD-L1 was inhibited in gene and protein level. The data are presented as the mean ± SEM. ***P < 0.001, **P < 0.01, (unpaired *t*-test)

Next, we investigated the mechanism of TRAIL-induced EMT. Programmed death-ligand 1 (PD-L1), as an immunosuppressive molecule, is highly expressed on the surface of a variety of cancer cells [23]. Recently, it was reported that PD-L1 promotes EMT, and thus induces tumor progression in lung and breast cancer [24–26]. To investigate whether PD-L1 is involved in TRAIL-induced EMT, we first analyzed the expression of immunosuppressive molecules (*CD274, CD279, TIM3, LAG3, CTLA4, CD38, CD101, MKI67, ICOS, EOMES, CD44, CD28, KLRG1, CD5, TIGIT, and CD47*) in the *TNFSF10 *high and low groups in TCGA database [27]. We found that *CD274* and *CTLA4* show significantly higher expression in the TNFSF10^high ^group than in the TNFSF10^low ^group (Fig. 5a); however, PD-L1 (*CD274*) was predominantly expressed by tumors [28] while CTLA4 and LAG3 were mostly expressed by T cells in the tumor microenvironment [29]. Notably, a markedly positive correlation was observed between *CD274 *and *TNFSF10* in ESCC tissues (Fig. 5b). To confirm this, we performed immunohistochemistry of ESCC samples collected from the patients. Similar expression results were obtained, and a significant correlation between PD-L1 and TRAIL was observed (Fig. 5c, Supplementary Fig. 3f). Furthermore, we examined TRAIL-knockdown and overexpressed cells and found that PD-L1 expression was lower in the knockdown group and higher in the overexpression group than in control (Fig. 5d). Thus, these results suggest that TRAIL regulates PD-L1 expression in ESCC.

We then assessed the mechanism underlying TRAIL regulation of PD-L1 expression. As a key molecule in tumor immune escape, the main mechanisms involved in PD-L1 regulation have been reported. We first examined the KEGG enrichment analysis in TCGA and found that only the MAPK/STAT3 signaling pathway was significantly up-regulated in tissues with high TRAIL expression (Fig. 5e). To reveal whether TRAIL-induced PD-L1 expression depends on MAPK/STAT3, we performed western blotting of TRAIL-knockdown and overexpressed ESCC cells. Knocking down TRAIL in KYSE150 cells markedly reduced p-ERK and p-STAT3 expression, while overexpression in EC1 cells significantly increased p-ERK and p-STAT3 levels (Fig. 5f). Furthermore, treatment of EC1 cells with ERK or STAT3 inhibitors reversed the TRAIL overexpression-induced increase in PD-L1 levels (Fig. 5g,h). In order to further study the mechanism of ERK/STAT3 regulating PD-L1, we knockdown STAT3 in EC1 cells overexpressing TRAIL. We observed the expression of PD-L1 at both gene level and protein level. The results showed that the expression of PD-L1 was downregulated after silencing STAT3 in EC1 cells (Fig. 5i).

When PD-L1 antibody was added to TRAIL-overexpression EC1 cells, only N-cadherin was significantly downregulated, while E-cadherin and vimentin showed no difference in mRNA levels (Supplementary Fig. 4a). However, after silencing PD-L1 in TRAIL-overexpression EC1 cells, both N-cadherin and vimentin were markedly downregulated, while E-cadherin expression did not change (Supplementary Fig. 4b). The aggressive and proliferation abilities of tumor cells were also inhibited (Supplementary Fig. 4c, e). These findings indicate that cytoplasmic PD-L1 promotes EMT, whereas PD-L1 expressed on the cell membrane cannot promote cell metastasis and invasion. Meanwhile, stemness-related genes and cell sphere-forming ability were detected and it found that the stemness of PD-L1 knockdown cells was reduced (Supplementary Fig. 4d, f-g), suggesting that PD-L1 also affects the stemness of ESCC cells. Altogether, the results demonstrate that TRAIL-induced EMT is dependent on ERK/STAT3-activated PD-L1 expression in ESCC.

### TRAIL activates the ERK/STAT3 pathway, inducing PD-L1 and promoting EMT of ESCC cells in vivo

**Fig. 6:**
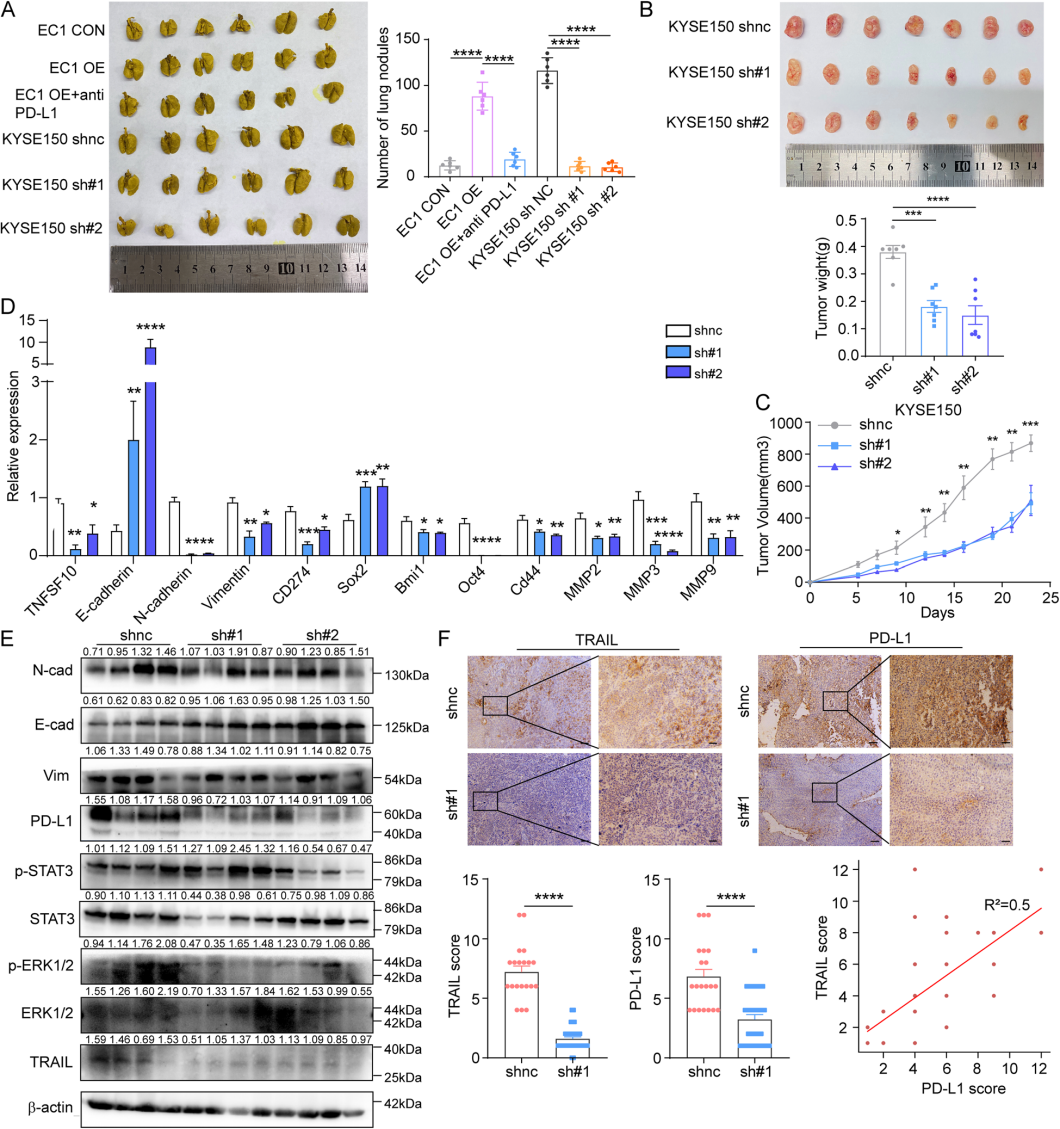
TRAIL promotes EMT of ESCC via PD-L1 in vivo. **a** TRAIL stably overexpression EC1 cells or knockdown KYSE150 cells were injected into the vein of SCID/Beige mice, and TRAIL overexpressing EC1 cells were intraperitoneally injected with anti-PD-L1 antibody. After 5 weeks, the lungs were harvested and the nodules were counted. **b, c** Tumor weight and volume were monitored every other day, mice were sacrificed after 21 days. Tumor growth was significantly inhibited in TRAIL-knockdown cells. **d, e** mRNA and protein levels of EMT and stemness markers in mouse tumor tissues. **e** Knockdown of TRAIL downregulated STAT3 and ERK phosphorylation levels in tumor tissues. **f** Immunohistochemistry of TRAIL and PD-L1 expression in animal tissues; immunohistochemical scoring and correlation analysis were performed. The data are presented as the mean ± SEM. ***P < 0.001, **P < 0.01, *P < 0.05 (unpaired *t*-test)

EMT is an essential event in the metastasis of many solid tumors, we next examined whether TRAIL promote metastasis in ESCC cells in vivo. We used an ESCC cells adoptive transferred mouse model to detect the effect of TRAIL expression or anti-PDL1 treatment on lung metastasis of ESCC in vivo. It showed that the number of lung nodules was significantly upregulated by TRAIL overexpression in EC1 cells and anti-PD-L1 antibody effectively inhibited the metastasis of ESCC. Moreover, TRAIL knockdown decreased the lung metastasis of KYSE150 cells (Fig. 6a). Next, we tested whether TRAIL promotes EMT through ERK/STAT3-activated PD-L1 expression in a mouse model. Knocking down TRAIL significantly delayed the tumor growth of KYSE150 cells (Fig. 6b, c). Moreover RT-qPCR and western blotting showed a significant increase in E-cadherin expression and a marked decrease in the expression of N-cadherin, vimentin, PD-L1, MMP2, MMP3, MMP9, and stemness-related genes (Sox2, Bmi1, Oct4, and Cd44) in shTRAIL-KYSE150 cells compared with control (Fig. 6d, e). Meanwhile, immunohistochemistry data showed a significant positive correlation between TRAIL and PD-L1 (Fig. 6f). In contrast, overexpression of TRAIL in EC1 cells significantly accelerated tumor growth (Supplementary Fig. 5a, b) and promoted EMT- and stemness-related gene expression at the mRNA and protein levels (Supplementary Fig. 5c, d). Moreover, overexpression of TRAIL upregulated p-ERK/p-STAT3/PD-L1 levels (Supplementary Fig. 5d). The correlation between TRAIL and PD-L1 was also demonstrated (Supplementary Fig. 5e–g). Taken together, these data indicate that TRAIL promotes EMT-induced cell metastasis through ERK/STAT3 signaling pathway activation and inducing PD-L1 expression in ESCC in vivo.

## Discussion

In this study, we identified an ERK/STAT3-dependent mechanism whereby TRAIL induces PD-L1 expression, which in turn promotes EMT and tumor progression in ESCC. The role of TRAIL in tumors is still poorly understood. Although TRAIL was initially considered as the important inflammatory cytokines, many clinical trials with recombinant human TRAIL or TRAIL receptor agonists have failed. TRAIL is also expressed in cancer cells and verified with the functions promoting tumor progression. However, the role of TRAIL in ESCC is poorly defined. Herein, we found that there was a higher expression of TRAIL in ESCC tumor tissues and it was negatively correlated with clinical parameters as well as overall survival. Furthermore, we found that TRAIL had a various expression patterns in ESCC cell lines, which induced EMT and stemness through the ERK/STAT3/PD-L1 pathway. These results highlight the unique features of TRAIL in ESCC.

To date, the role of TRAIL in cancer remains controversial, as its function varies depending on cell type. Initially, as a secondary transmembrane protein, TRAIL was reported to be mainly expressed on the surface of immune cells and involved in the immune response. However, the diversity of TRAIL functions was gradually revealed. Singh et al. [30] reported that the cytotoxic effects of T cells predominantly rely on the expression of TRAIL on their surface. Several studies have shown that TRAIL promotes apoptosis in cancer cells [31–33]. These results indicate that TRAIL plays an antitumor role in the tumor microenvironment. Interestingly, a few studies reported that TRAIL can promote tumor invasion and metastasis in tumors with KRAS mutations [9, 34, 35], and TRAIL promotes NF-kB-dependent tumor cell migration and invasion without influencing proliferation [36]. TRAIL was also shown to promote the immunosuppressive cancer microenvironment by inducing Treg proliferation [37, 38]. In addition, TRAIL can induce pro-inflammatory cytokines to polarize myeloid cells toward myeloid-derived suppressor cells and complete differentiation of the M2 macrophage phenotype [9, 39]. However, the main source of TRAIL and its cytological function in esophageal cancer is still not well understood. Herein, we found that TRAIL was mainly produced by tumor cells and promoted the metastasis or invasion in ESCC. For the first time, we revealed the biological role of TRAIL in ESCC, expanding the diversity of TRAIL functions. Both tumor cells and immune cells can express TRAIL, but they have different functions; however, we did not perform further studies on this functional difference. Based on the reported studies, these discrepant functions may be due to: (1) the different states of the tumor microenvironment; (2) structural differences between these two different sources of TRAIL; and (3) different concentrations of TRAIL in the tumor microenvironment. Indeed, it has been reported that low doses of TRAIL promote tumor cell invasion and metastasis by activating p-STAT3 in non-small cell lung cancer [4, 40] and that TRAIL induces a significant amount of apoptosis in a human T-cell line in a dose-dependent manner [41].

EMT is a key process in tumor invasion and metastasis. Various factors contribute to EMT, including low oxygen, low pH acidic environment, stromal cells, and tumor cells, as well as the secretion of various inflammatory factors and cytokines by immune cells [12, 42]. In recent years, immune checkpoint inhibitors have also been reported to be associated with EMT, especially PD-L1. It was reported that PD-L1 promotes EMT and carcinogenesis in pancreatic cancer [15]. Additionally, the role of PD-L1 in regulating EMT has also been reported in lung cancer [14, 43] and esophageal cancer [13]. Consistent with previous studies, we demonstrated that TRAIL-mediated EMT in ESCC is dependent on PD-L1 expression in the cytoplasm.

Some studies have reported that TRAIL and PD-L1 expression is consistent in lung and gastric cancer [17, 44, 45], but there are no relevant studies exploring their mechanisms. Mitogen activated protein kinases (MAPKs) are a class of serine/threonine protein kinases. The MAPK signal transduction pathway is present in most cells and plays a crucial role in transducing extracellular signals to the intracellular nucleus, causing biological responses such as cell proliferation, differentiation, and transformation [46]. Three parallel MAPK signaling pathways have been identified in cells: (1) the ERK pathway, which is considered the classical MAPK signaling pathway; (2) the JNK/SAPK pathway; and (3) the p38MAPK pathway [47]. STAT3, signal transducer and activator of transcription 3, plays an important role in the progression of multiple tumors. It has also been reported that the upregulation of MAPK can specifically promote the phosphorylation of a serine (Ser) at the C-terminus of STAT3, which greatly enhances the transcriptional activity of STAT3 [48]. It has also been reported that ERK1/2 and STAT3 increase the resistance of tumor cells to TRAIL-promoted apoptosis [49]. In our study, we found for the first time that TRAIL can promote PD-L1 expression through the ERK/STAT3 signaling pathway and promote EMT, as evidenced by TCGA database and western blotting. Hence, we speculate that STAT3/ERK inhibitors can be combined with TRAIL recombinant proteins to treat tumors, which warrants further studies.

In conclusion, although TRAIL is known to be expressed in immune cells, this is the first report to reveal that the source of TRAIL expression in ESCC cells. Furthermore, we investigated the correlation between TRAIL and PD-L1 and found that TRAIL upregulates PD-L1 expression through the STAT3/ERK signaling pathway, further inducing EMT and tumor progression in ESCC. When we used anti-PD-L1 treatment, the metastasis was inhibited in a mouse model. (Supplementary Fig. 5g).

## Conclusions

In this study, we found that TRAIL was a poor predictor in patients with ESCC. Further research found that TRAIL was expressed in ESCC cells and induced EMT by upregulating PD-L1 expression, meanwhile the process was activated through the ERK/STAT3 signaling pathway. In addition, anti-PD-L1 antibody inhibited TRAIL-mediated metastasis in mice. Thus, TRAIL has the potential to act as a key molecule to target EMT and inhibit ESCC progression in the future.

## Supplementary Information

**Additional file 1: Supplementary Figure 1. **Silencing TRAIL downregulates invasion, proliferation, and stemness of ESCC. (a) TRAIL expression in different stages of tumors. (b) TRAIL knockdown efficiency by RT-qPCR. (c–e) TRAIL knockdown reduced invasion, spheroid, and proliferation abilities of ESCC cells. (f) Stemness marker mRNA levels in TRAIL-knockdown cell lines. Data are presented as the mean ± SEM, analyzed by unpaired t-test, *p<0.05, **p<0.01, ***p<0.001. **Supplementary Figure 2.** Recombinant human TRAIL (rh-TRAIL) upregulates ESCC stemness and invasion. (a, b) Flow cytometry of CD271 and CXCR4 expression in KYSE70 and KYSE150 cells. (c) mRNA levels of Bmi1 after addition of rh-TRAIL in ESCC cells. (d, e) Sphere formation and Transwell assays after addition of rh-TRAIL. Data are presented as the mean ± SEM, analyzed by unpaired t-test, *p<0.05, **p<0.01, ***p<0.001. **Supplementary Figure 3.** TRAIL promotes EMT progression in ESCC cells. (a, b) mRNA and protein levels of EMT-related markers in TRAIL-knockdown cell lines. (c, d) Expression of EMT-related markers after addition of rh-TRAIL. (e) Correlation analysis of E-cad, N-cad, Vim, PD-L1 and TRAIL in tissue microarrays. (f) Expression of TRAIL and PD-L1 in tumor tissues. Data are presented as the mean ± SEM, analyzed by unpaired t-test, *p<0.05, **p<0.01. **Supplementary Figure 4.** PD-L1 in the cytoplasm facilitates EMT. (a) EMT-related gene expression in vitro after addition of PD-L1 monoclonal antibody. (b) Silencing PD-L1 suppressed mRNA levels of N-cadherin and vimentin. (c, e, f) Silencing PD-L1 reduced the proliferation, invasion and spherical capacity of EC1 cells. (d, g) Silencing PD-L1 reduced the stemness capacity of tumor. Data are presented as the mean ± SEM, analyzed by unpaired t-test, *p<0.05, **p<0.01, ***p<0.001. **Supplementary Figure 5.** TRAIL promotes EMT of ESCC cells in vivo. (a, b) Tumor weight and volume were monitored, after which mice were sacrificed 21 days later. Tumor growth was significantly promoted in TRAIL-overexpression cells. (c, d) mRNA and protein levels of EMT and stemness markers in mouse tumor tissues. (d) Overexpression of TRAIL promoted STAT3 and ERK phosphorylation levels in tumor tissues. (e, f) Immunohistochemistry of TRAIL and PD-L1 expression in animal tissues; immunohistochemical scoring and correlation analysis were performed. (g) Mechanism diagram of TRAIL upregulating PD-L1 and promoting EMT progression in ECSS. Data are presented as the mean ± SEM, analyzed by unpaired t-test, *p<0.05, **p<0.01.

**Additional file 2.**

## Data Availability

The data sets used and analyzed during the current study are available from the corresponding author on reasonable request.

## Abbreviations

TRAIL: Tumor necrosis factor-associated apoptosis-inducing ligand
TNFSF: Tumor necrosis factor superfamily
OPG: Osteoprotegerin
ESCC: Esophageal squamous cell carcinoma
EMT: Epithelial-mesenchymal transition
PD-L1: Programmed death-ligand 1
MMPs: Matrix Metalloproteinases
siRNA: Small interfering RNA
rh-TRAIL: Recombinant human TRAIL
STAT3: Signal transducer and activator of transcription 3
MAPKs: Mitogen activated protein kinases
shRNAs: Short hairpin RNAs
PBS: Phosphate buffered saline
RT-qPCR: Quantitative real-time polymerase chain reaction
SD: Standard deviation
