# Supplementary material for: TRAIL promotes epithelial-to-mesenchymal transition by inducing PD-L1 expression in esophageal squamous cell carcinomas
Source: J Exp Clin Cancer Res. 2021 Jun 24;40:209. doi: 10.1186/s13046-021-01972-0 (PMC8223376; doi:10.1186/s13046-021-01972-0)
Supplement: Supplementary file 1 — Additional file 1: Supplementary Figure 1. Silencing TRAIL downregulates invasion, proliferation, and stemness of ESCC. (a) TRAIL expression in different stages of tumors. (b) TRAIL knockdown efficiency by RT-qPCR. (c–e) TRAIL knockdown reduced invasion, spheroid, and proliferation abilities of ESCC cells. (f) Stemness marker mRNA levels in TRAIL-knockdown cell lines. Data are presented as the mean ± SEM, analyzed by unpaired t-test, *p<0.05, **p<0.01, ***p<0.001. Supplementary Figure 2. Recombinant human TRAIL (rh-TRAIL) upregulates ESCC stemness and invasion. (a, b) Flow cytometry of CD271 and CXCR4 expression in KYSE70 and KYSE150 cells. (c) mRNA levels of Bmi1 after addition of rh-TRAIL in ESCC cells. (d, e) Sphere formation and Transwell assays after addition of rh-TRAIL. Data are presented as the mean ± SEM, analyzed by unpaired t-test, *p<0.05, **p<0.01, ***p<0.001. Supplementary Figure 3. TRAIL promotes EMT progression in ESCC cells. (a, b) mRNA and protein levels of EMT-related markers in TRAIL-knockdown cell lines. (c, d) Expression of EMT-related markers after addition of rh-TRAIL. (e) Correlation analysis of E-cad, N-cad, Vim, PD-L1 and TRAIL in tissue microarrays. (f) Expression of TRAIL and PD-L1 in tumor tissues. Data are presented as the mean ± SEM, analyzed by unpaired t-test, *p<0.05, **p<0.01. Supplementary Figure 4. PD-L1 in the cytoplasm facilitates EMT. (a) EMT-related gene expression in vitro after addition of PD-L1 monoclonal antibody. (b) Silencing PD-L1 suppressed mRNA levels of N-cadherin and vimentin. (c, e, f) Silencing PD-L1 reduced the proliferation, invasion and spherical capacity of EC1 cells. (d, g) Silencing PD-L1 reduced the stemness capacity of tumor. Data are presented as the mean ± SEM, analyzed by unpaired t-test, *p<0.05, **p<0.01, ***p<0.001. Supplementary Figure 5. TRAIL promotes EMT of ESCC cells in vivo. (a, b) Tumor weight and volume were monitored, after which mice were sacrificed 21 days later. Tumor growth was signifi [file 13046_2021_1972_MOESM1_ESM.docx]

**Supplementary Figure**


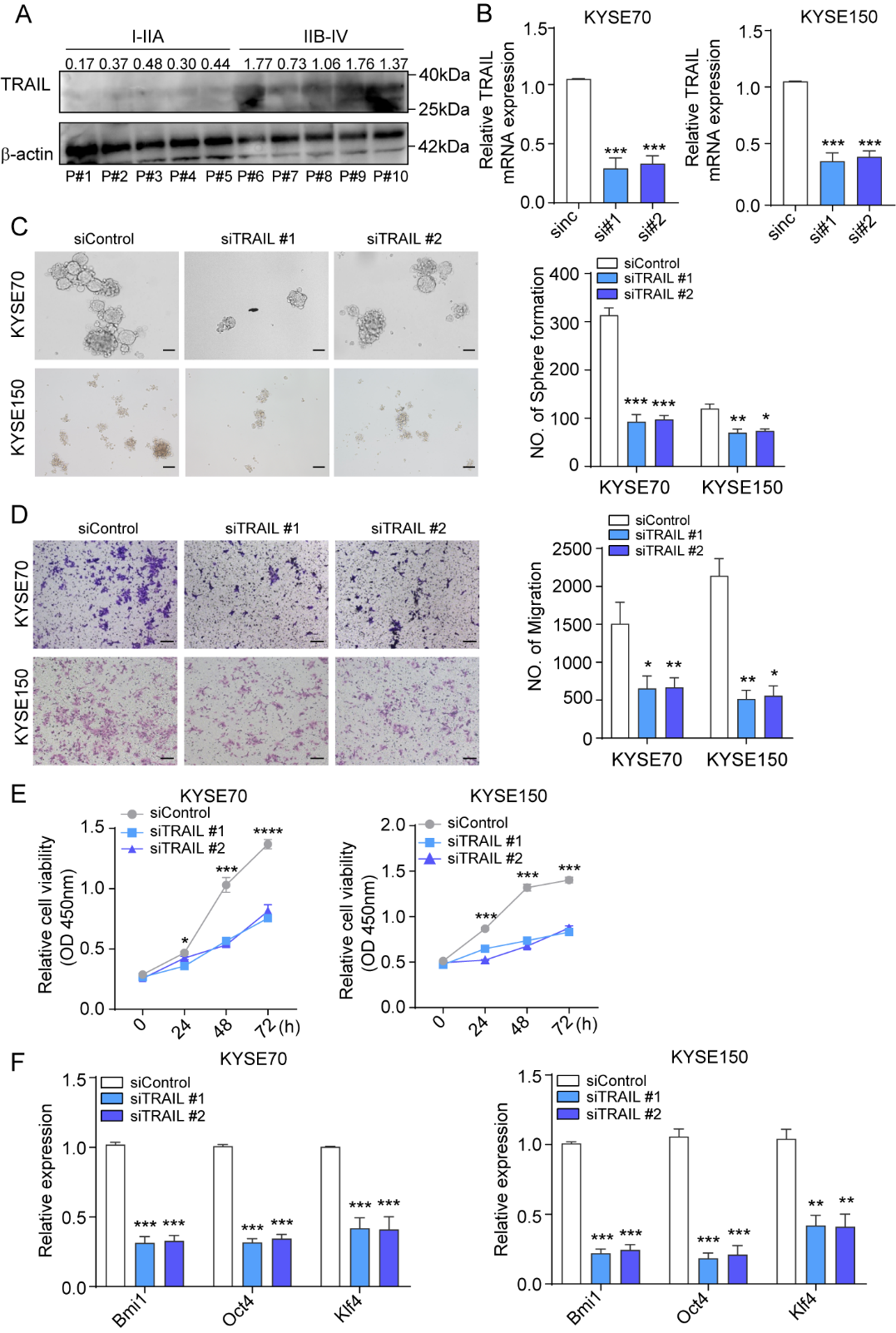


**Supplementary Figure 1. Silencing TRAIL downregulates invasion, proliferation, and stemness of ESCC.** (a) TRAIL expression in different stages of tumors. (b) TRAIL knockdown efficiency by RT-qPCR. (c–e) TRAIL knockdown reduced invasion, spheroid, and proliferation abilities of ESCC cells. (f) Stemness marker mRNA levels in TRAIL-knockdown cell lines. Data are presented as the mean ± SEM, analyzed by unpaired t-test, *p<0.05, **p<0.01, ***p<0.001.


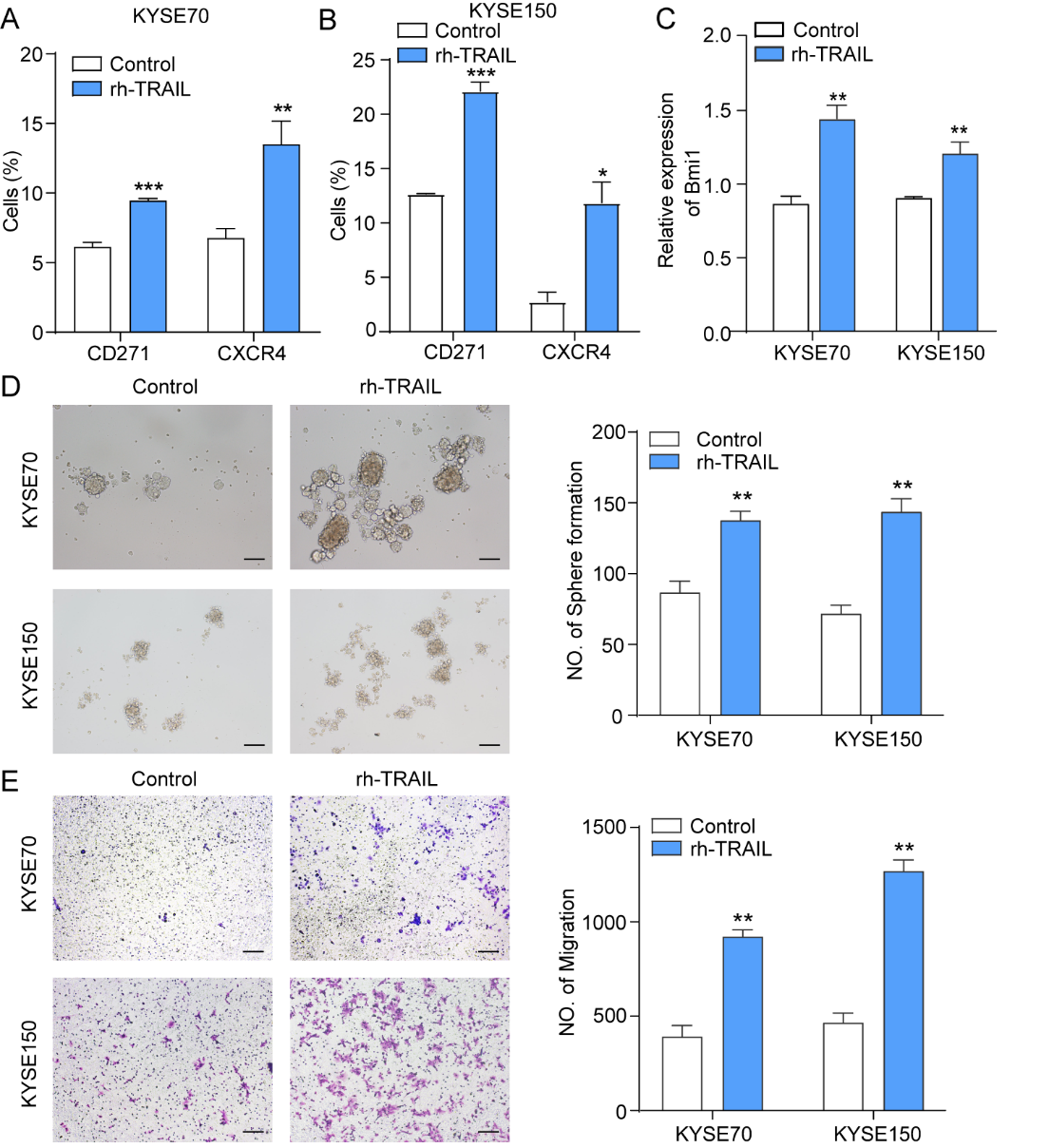


**Supplementary Figure 2. Recombinant human TRAIL (rh-TRAIL) upregulates ESCC stemness and invasion.** (a, b) Flow cytometry of CD271 and CXCR4 expression in KYSE70 and KYSE150 cells. (c) mRNA levels of Bmi1 after addition of rh-TRAIL in ESCC cells. (d, e) Sphere formation and Transwell assays after addition of rh-TRAIL. Data are presented as the mean ± SEM, analyzed by unpaired t-test, *p<0.05, **p<0.01, ***p<0.001.


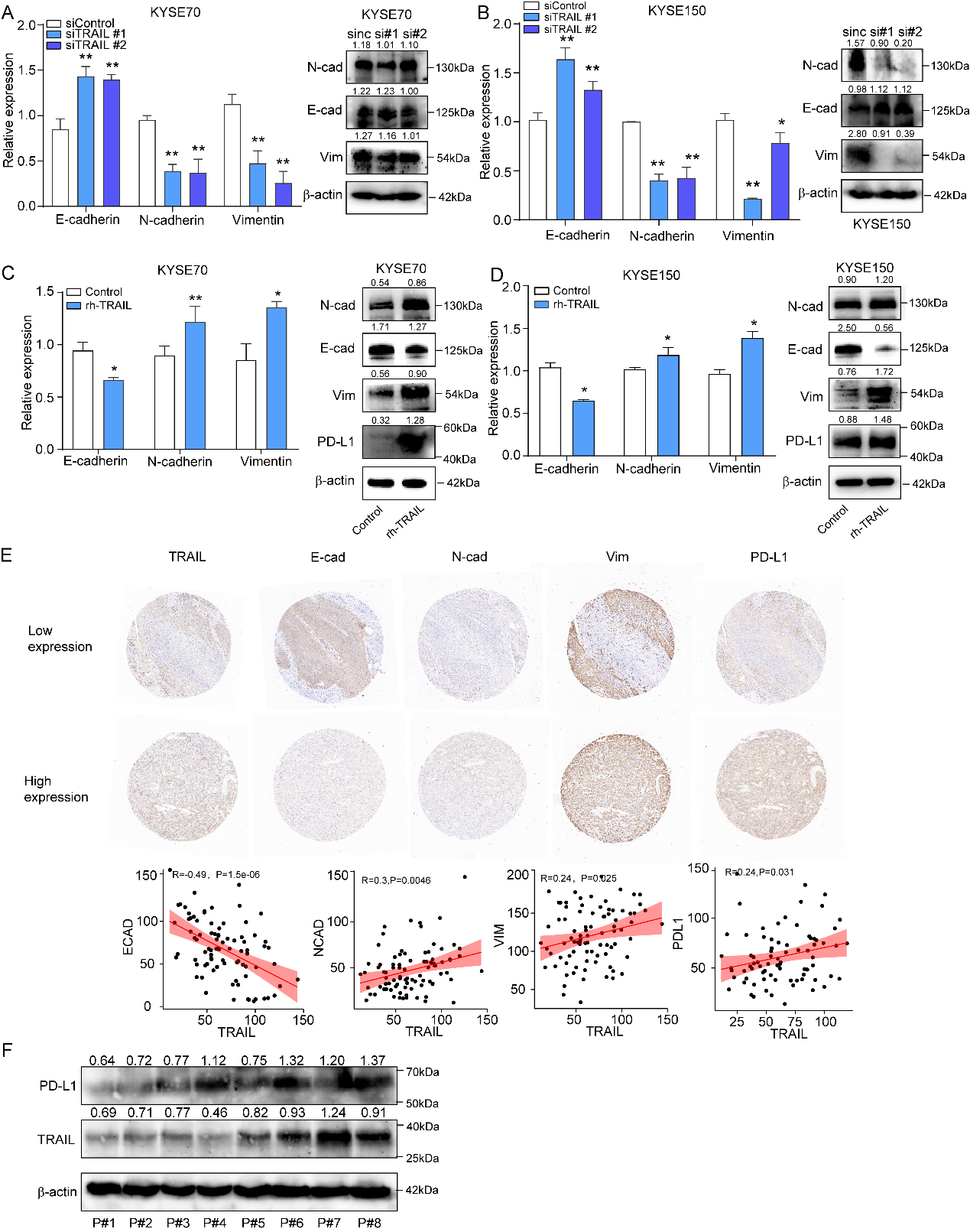


**Supplementary Figure 3. TRAIL promotes EMT progression in ESCC cells.** (a, b) mRNA and protein levels of EMT-related markers in TRAIL-knockdown cell lines. (c, d) Expression of EMT-related markers after addition of rh-TRAIL.

(e) Correlation analysis of E-cad, N-cad, Vim, PD-L1 and TRAIL in tissue microarrays. (f) Expression of TRAIL and PD-L1 in tumor tissues. Data are presented as the mean ± SEM, analyzed by unpaired t-test, *p<0.05, **p<0.01.


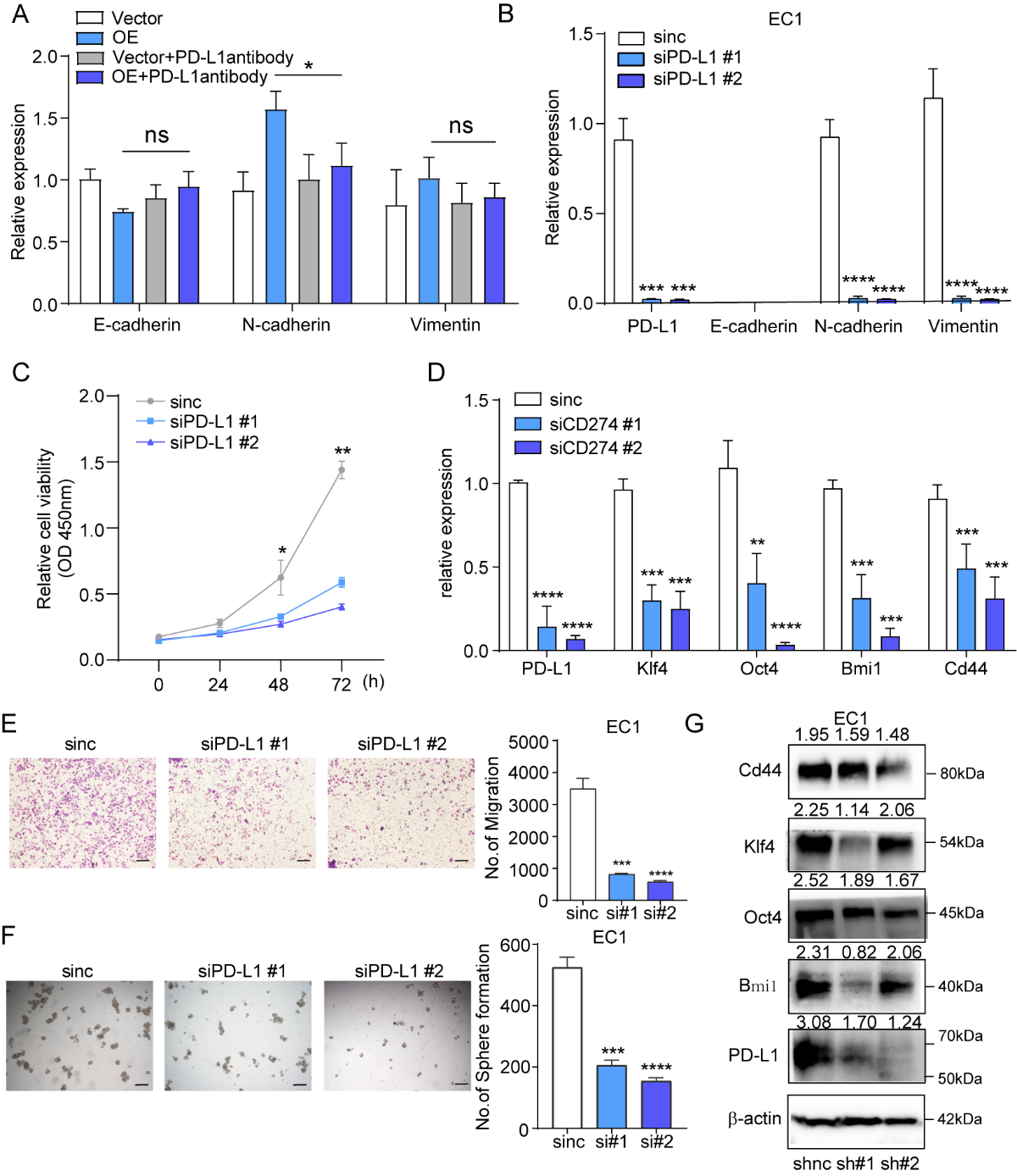


**Supplementary Figure 4. PD-L1 in the cytoplasm facilitates EMT.** (a) EMT-related gene expression in vitro after addition of PD-L1 monoclonal antibody. (b) Silencing PD-L1 suppressed mRNA levels of N-cadherin and vimentin. (c, e, f) Silencing PD-L1 reduced the proliferation, invasion and spherical capacity of EC1 cells. (d, g) Silencing PD-L1 reduced the stemness capacity of tumor. Data are presented as the mean ± SEM, analyzed by unpaired t-test, *p<0.05, **p<0.01, ***p<0.001.


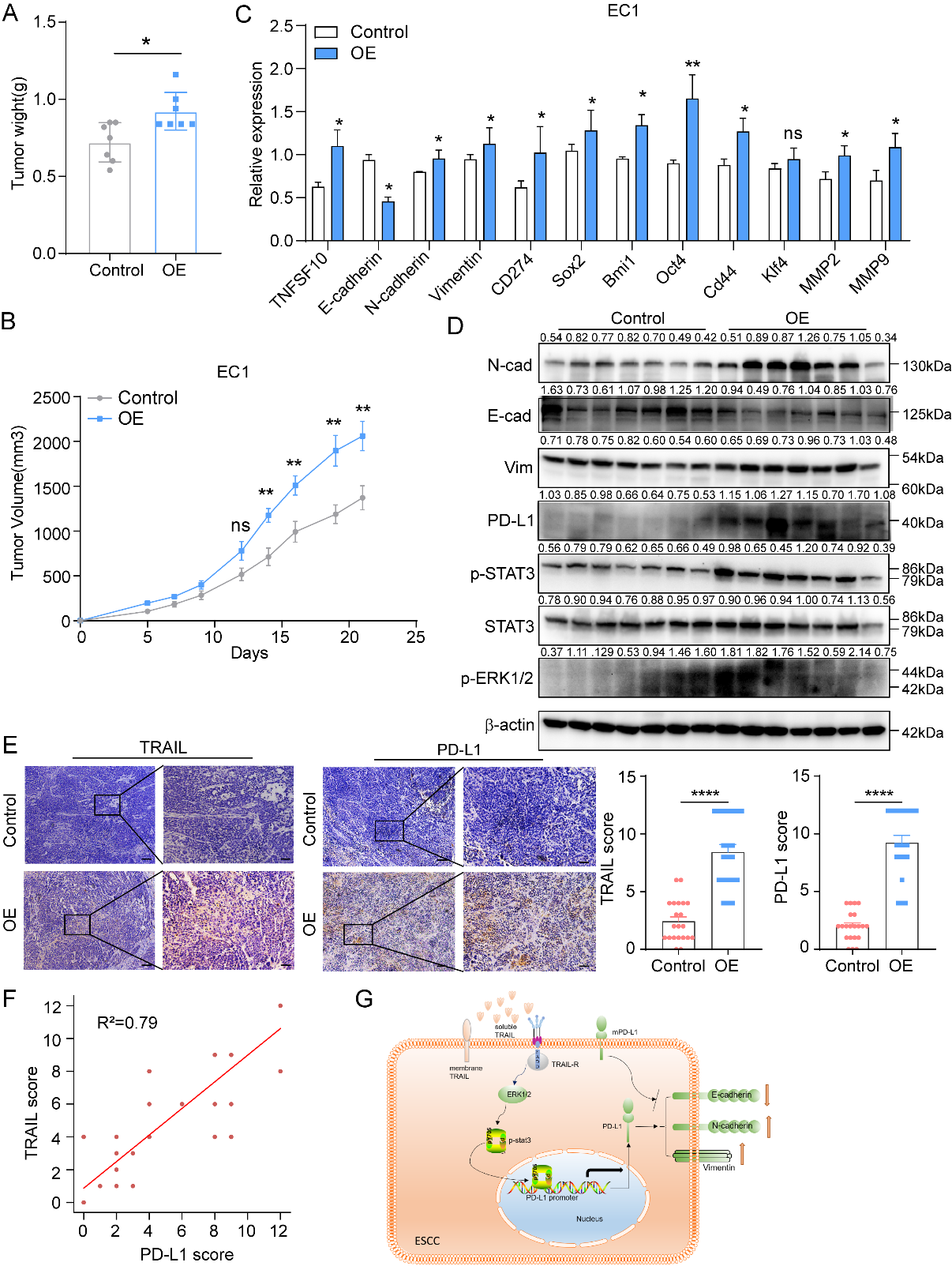


**Supplementary Figure 5. TRAIL promotes EMT of ESCC cells in vivo.** (a, b) Tumor weight and volume were monitored, after which mice were sacrificed 21 days later. Tumor growth was significantly promoted in TRAIL-overexpression cells. (c, d) mRNA and protein levels of EMT and stemness markers in mouse tumor tissues. (d) Overexpression of TRAIL promoted STAT3 and ERK phosphorylation levels in tumor tissues. (e, f) Immunohistochemistry of TRAIL and PD-L1 expression in animal tissues; immunohistochemical scoring and correlation analysis were performed. (g) Mechanism diagram of TRAIL upregulating PD-L1 and promoting EMT progression in ECSS. Data are presented as the mean ± SEM, analyzed by unpaired t-test, *p<0.05, **p<0.01.
