# Supplementary material for: TRAIL promotes epithelial-to-mesenchymal transition by inducing PD-L1 expression in esophageal squamous cell carcinomas
Source: J Exp Clin Cancer Res. 2021 Jun 24;40:209. doi: 10.1186/s13046-021-01972-0 (PMC8223376; doi:10.1186/s13046-021-01972-0)
Supplement: Supplementary file 2 — Additional file 2. [file 13046_2021_1972_MOESM2_ESM.docx]

Supplementary Table 1

| REAGENT or RESOURCE | SOURCE | IDENTIFIER |
| --- | --- | --- |
| Antibodies | | |
| TRAIL (C92B9) Rabbit mAb | Cell Signaling Technology | #3219 |
| Phospho-p44/42MAPK(Erk1/2)(Thr202/Tyr204)(D13.14.4E) Rabbit mAb | Cell Signaling Technology | #4370 |
| p44/42 MAPK (Erk1/2) (137F5) Rabbit mAb | Cell Signaling Technology | #4695 |
| Stat3 (D3Z2G) Rabbit mAb | Cell Signaling Technology | #12640 |
| Phospho-Stat3 (Tyr705) (D3A7) Rabbit mAb | Cell Signaling Technology | #9145 |
| β-Actin (8H10D10) Mouse mAb | Cell Signaling Technology | #3700 |
| E-Cadherin (24E10) Rabbit mAb | Cell Signaling Technology | #3195 |
| N-Cadherin (D4R1H) XP^®^ Rabbit mAb | Cell Signaling Technology | #13116 |
| Vimentin (D21H3) XP^®^ Rabbit mAb | Cell Signaling Technology | #5741 |
| CD44 (E7K2Y) XP® Rabbit mAb | Cell Signaling Technology | #37259 |
| Anti-Oct4 Rabbit mAb [EPR17929] | abcam | ab181557 |
| Anti-KLF4 Rabbit mAb [EPR19590] | abcam | ab215036 |
| Anti-Bmi1 Rabbit mAb [EPR3745(2)] | abcam | ab126783 |
| Anti-PD-L1 Rabbit mAb | abcam | ab213524 |
| E-Cadherin Monoclonal Antibody | proteintech | 60335-1-Ig |
| Vimentin Polyclonal Antibody | proteintech | 10366-1-AP |
| N-Cadherin Polyclonal Antibody | proteintech | 22018-1-AP |
| PerCP/Cyanine5.5 anti-human CD271 (NGFR) Antibody | Biolegend | Cat# 345111 |
| APC anti-human CD184 (CXCR4) Antibody | Biolegend | Cat# 306509 |
| Chemical,Peptides,and Recombinant Proteins | | |
| C188-9 | MCE | Cat# HY-112288 |
| ERK1/2 inhibitor 1 | MCE | Cat# HY-112287 |
| Recombinant Human TRAIL | Biolegend | Cat# 752906 |
| Primers |  |  |
| GAPDH-F | ACAACTTTGGTATCGTGGAAGG |  |
| GAPDH-R | GCCATCACGCCACAGTTTC |  |
| TNFSF10-F | GAAGCAACACATTGTCTTCTCCAA |  |
| TNFSF10-R | TTGCTCAGGAATGAATGCCC |  |
| E-cadherin-F | TGCACCAACCCTCATGAGTG |  |
| E-cadherin-R | GTCAGTATCAGCCGCTTTCAG |  |
| N-cadherin-F | ACAGTGGCCACCTACAAAGG |  |
| N-cadherin-R | CCGAGATGGGGTTGATAATG |  |
| Vimentin-F | GACGCCATCAACACCGAGTT |  |
| Vimentin-R | CTTTGTCGTTGGTTAGCTGGT |  |
| Klf4-F | CCCACATGAAGCGACTTCCC |  |
| Klf4-R | CAGGTCCAGGAGATCGTTGAA |  |
| Bmi1-F | CCACCTGATGTGTGTGCTTTG |  |
| Bmi1-R | TTCAGTAGTGGTCTGGTCTTGT |  |
| Cd44- F | TCAGAGGAGTAGGAGAGAGGAAAC |  |
| Cd44-R | GAAAAGTCAAAGTAACAATAACAGTGG |  |
| Sox2- F | CGAGTGGAAACTTTTGTCGGA |  |
| Sox2- R | TGTGCAGCGCTCGCAG |  |
| Oct4-F | GTGGAGAGCAACTCCGATG |  |
| Oct4-R | TGCTCCAGCTTCTCCTTCTC |  |
| Plasmids | | |
| Negative control | UUCUCCGAACGUGUCACGUTT | genepharma |
| si-TNFSF10-1 | UGGCUACUAUUAGAUGACCAGUAAA | genepharma |
| si-TNFSF10-2 | CCUGGGUAAUAAUGUAGCUACAUUA | genepharma |
| si-CD274-1 | GUGGCAUCCAAGAUACAAATT | genepharma |
| si-CD274-2 | CUGGGAGCCAUCUUAUUAUTT | genepharma |
| si-STAT3-1 | GGGACCUGGUGUGAAUUAUTTAUAAUUCACACCAGGUCCCTT | genepharma |
| si-STAT3-2 | GGUACAUCAUGGGCUUUAUTTAUAAAGCCCAUGAUGUACCTT | genepharma |
| TNFSF10-RNAi (19778-1) | gtAACAAATGAGCACTTGATA | genechem |
| TNFSF10-RNAi (19779-1) | cgACAAACAAATGGTCCAATA | genechem |
